# Abstract of the Proceedings of the Buffalo Medical Association

**Published:** 1857-03

**Authors:** Sanford B. Hunt


ART. VII.—*Abstract of the Proceedings of the Buffalo Medical Association.*

TUESDAY EVENING, Feb. 2, 1857.

The association met.

Present—The Vice-president, Dr. Flint, in the chair. Drs. Newman, Hadley, Underhill, Nichols, Root, Hawley, Hunt, Wilcox, Strong, Rogers, Jeyte, Dayton, Mixer and Gay.

The minutes of the preceding meeting were read and approved.

*Dr. Root* mentioned the case of a lady, suffering from remittent fever, to whom he was called after she had been for a fortnight under the care of a homœopath. He found her with a sore mouth, breath having mercurial fœtor, and gums and tongue presenting the usual appearances of mercurial ptyalism. The homœopath denied having given any mercury, but said that she was bilious, and he had endeavored to drive the bile from the liver into the stomach.

Many members mentioned cases of scarlatina; *Dr. Mixer* one which was taken with convulsions, and died in four hours after the attack.

*Prof. Hadley* presented a specimen of a new cosmetic, much vaunted, called "Kaliston, or Orient Water." It was very neatly put up, accompanied by a pamphlet essay on beauty, recommending it for tan, sunburn, freckles, pimples, etc. It is largely advertised. In appearance it is a clear fluid, scented with rose; has a slight sediment and not much taste. It contains alcohol enough to prevent its freezing. A chemical analysis shows that it contains mercury, ammonia, and chlorine. The inferences are that it is a solution of corrosive sublimate, the muriate of ammonia being used to conceal it. The quanitty of the poison is probably too small to produce much injurious effects.

The same gentleman also presented a specimen of granulated sugar, sold for frosting cakes. It was of a green color. It consists of sugar, colored with Paris green, an irregular compound of arsenite of copper and acetate of copper, in the ratio of one per cent. A child eating an ounce of this would take nearly five grains of this poisonous compound. Prof. H. had known a whole family poisoned and made very sick by this article, procured from a druggist.

*Prof. Flint* inquired if members had seen cases of acute pharyngitis, accompanied with sharp febrile movement. It seemed to be associated with scarlatina, in some cases the character of which was doubtful. Adults were usually confined to bed with it for two or three days. These cases were diptheritic, and were probably rare here, though the French say much about diptheritic pharyngitis. He had heard Trousseau speak of the difficulty in diagnosticating it from scarlatina. It was of this disease that Valliex died.

*Dr. Strong* stated that he had seen adults with tonsillitis and pharyngitis combined, which he was inclined to believe had communicated scarlatina to children.

*Dr. Wilcox* had had a case of a lad of sixteen, who had tonsillitis only, but following his attack three or four other children in the same family had scarlatina.

*Prof. Flint* gave details of a case of asthma. The patient was admitted to the hospital with asthma, accompanied by moderate emphysema, and not much bronchitis. He received remarkable relief from the inhalation of chloroform. The paroxysms occurred once a week, pure spasm being the chief element. The chloroform, while it did not prevent the recurrence of the paroxysm, cut it short and very much relieved it.

Prof. F. also reported the case of a patient entering the hospital a week before, complaining of want of breath on exercise. The physical signs were dilation, immobility and tympany of the left side. There was no respiratory murmur, and the case was diagnosticated as emphysema. The next day he had metallic respiration and voice, and on the day next succeeding, well-marked metallic tinkling was heard beneath and behind the scapula. He had no pain or fever; was generally healthy.

The history was as follows:

A year or two before, he had some pulmonic disease, the character of which is unknown. He was a pedler, and some twelve days before entering the hospital he had been obliged to put down his pack, weighing some eighty pounds, on account of severe pain under the scapula. He had probably had local emphysema before, and at that time rupture, and general emphysema followed. There was no evidence of any effusion, and this renders the metallic tinkling remarkable.

The distention is now diminishing.

And the association then adjourned.

SANFORD B. HUNT, Secretary.

